# Microcephaly, Short Stature, Intellectual Disability, Speech Absence and Cataract Are Associated with Novel Bi-Allelic Missense Variant in *RTTN* Gene: A Seckel Syndrome Case Report

**DOI:** 10.3390/children10061027

**Published:** 2023-06-08

**Authors:** Behjat Ul Mudassir, Zehra Agha

**Affiliations:** Translational Genomics Laboratory, Department of Biosciences, COMSATS University, Islamabad 45550, Pakistan; zehra.agha@comsats.edu.pk

**Keywords:** Seckel syndrome case in Islamabad, *RTTN* gene related Seckel syndrome case, missense variant in *RTTN* gene, MCPH and *RTTN* gene missense mutation

## Abstract

The *RTTN* gene encodes centriole biogenesis, replication, symmetry and cohesion, basal body organization and has recently been associated with the appearance of microcephaly syndromes. *RTTN*-related neurological defects including microcephaly, intellectual disability, congenital dwarfism, ophthalmic manifestations, and epilepsy are mainly due to abnormal brain development pathways and loss-of-function protein mutations. We present a consanguineous Pakistani family clinically suspected of Seckel syndrome with severe microcephaly, severe intellectual disability, short stature, absence of speech, pointed nose, narrow face and bilateral cataract in two siblings residing in the suburbs of Islamabad. Forty cases of Seckel syndrome have been reported to date in the literature due to mutations in the *ATR*, *TRAIP*, *RBBP8*, *NSMCE2*, *NIN*, *CENPJ*, *DNA2, CEP152* and *CEP63*  genes. The objective of the study was to perform a clinical diagnosis, genetic analysis, and pathophysiology of Seckel syndrome in the proband. Whole-exome sequencing discovered NM_173630.4: c.57G > T(pGlu19Asp) missense variant in exon 2 of the *RTTN* gene that co-segregates in the family. This novel variant, to the best of our knowledge, is pathogenic and with autosomal recessive inheritance expressed as Seckel syndrome in the affected members of the family. The present study has expanded the genetic knowledge of novel *RTTN* gene variants associated with Seckel syndrome and has broadened its phenotype spectrum in the Pakistani population, which comprises diverse ethnicities. We hope that our study will open new horizons for individual molecular diagnosis and therapeutics to improve the life of patients with this congenital syndrome.

## 1. Introduction

Seckel syndrome is a very rare, congenital, autosomal recessive and distinct genetic condition [1] in which the circumference of the frontal and occipital skull is reduced due to prenatal neural growth abnormalities [2] and distinct facial features such as a pointed nose and narrow face appear in patients with short stature and intellectual disability [3]. Co-morbid clinical features include ophthalmic manifestations [4], cardiac abnormalities [5] and dental anomalies. Mann and Russell originally reported Seckel syndrome in 1959, and Helmut Paul George Seckel reported this syndrome in detail in 1960 for the first time; since then, very few additional cases have been reported [6]. The diagnosis of this syndrome is often difficult due to variable phenotypes, and it mostly requires genetic or molecular analysis [7,8]. *ATR*, *TRAIP*, *RBBP8*, *NSMCE2*, *NIN*, *CENPJ*, *DNA2, CEP152* and *CEP63*  genes have been found to be involved in the phenotype of this syndrome so far. Many families diagnosed with syndromic-autosomal recessive primary microcephaly (MCPH) do not carry variants in these genes; hence, more research is required to broaden the genetic and phenotypic spectrum [9]. Most of the MCPH-related genes encode proteins that control centriole formation, proliferation and maturation during cell division [10]. The current explosion of disease-gene findings, however, has shown more commonalities than distinctions in the underlying genomic pattern for many clinical sub-categories [11]. Microcephaly syndromes have been reported for the *ASPM*, *PHC1*, *STIL*, *MCPH1*, *WDR62*, *CDK5RAP2*, *CIT*, *CENPJ* and *KNL1* genes, which are associated with short stature, MCPH, ophthalmic, auditory and dental anomaly phenotypes [11]. Intellectual disability, developmental delay, cerebral defects, intrauterine growth restrictions, abnormal neuro-motor development, abnormal synaptic patterns and post-natal dysmorphic facial feature defects have been observed in patients with syndromic forms of MCPH [12]. Differential diagnosis for MCPH and its variable penetrance and expressivity in neural and extra-neural events has been led by the refined genetic orifice, which is targeted for the evaluation of microcephaly phenotype [13]. The decrease in cerebral volume, particularly for the cerebral cortex, due to syndromic microcephaly has been connected primarily to abnormalities in the growth and survival of brain cell precursors. Likewise, there are deviations in synaptic development that interfere with action potentials and ionic balance [14]. These malformations associated with primary microcephaly are mainly encoded by the twenty-eight gene variations reported so far [15].

Here, we report the role of an exon 2 of the *RTTN* missense variant in Seckel syndrome for the first time in the Pakistani population. Rotatin (RTTN) is a centrosome-associated protein expressed by *RTTN,* and its loss impairs centriole integrity, biogenesis and embryonic lethality [16]. *RTTN* mutations have recently been reported for MCPH, short stature, skin atrophy and brachydactyly in families with neurodevelopmental malformations due to mitotic arrest and disruption in cell cycle progression [17,18]. Gene dosage alterations as a result of gain or loss in gene function due to overexpression or under expression of rotatin protein cause disruptions to cell cycle and apoptosis regulation and neurological dysfunction [19]. ClinVar contains twenty-nine pathogenic and three likely pathogenic variants in multiple exons of the *RTTN* gene with microcephaly, dwarfism, seizures, development delay and polymicrogyria. Frameshift, missense, nonsense and splice site variants have been reported in the ClinVar database for the *RTTN* gene and MCPH phenotypes, which presents this gene as a strong candidate for genetic and molecular characterization of congenital microcephaly (accessed March 2023). This study presents a family with a previously unreported clinical diagnosis of microcephaly, intellectual disability, short stature, seizures, absence of speech, retinal lesions, bilateral cataract, and myelin dystrophy as revealed in an MRI of two affected siblings born to normal parents with a consanguineous marriage. Whole-exome sequencing disclosed a novel, pathogenic, bi-allelic, missense mutation in the *RTTN* gene (exon 2).

## 2. Case Presentation

### 2.1. Clinical Diagnosis

**Figure 1:**
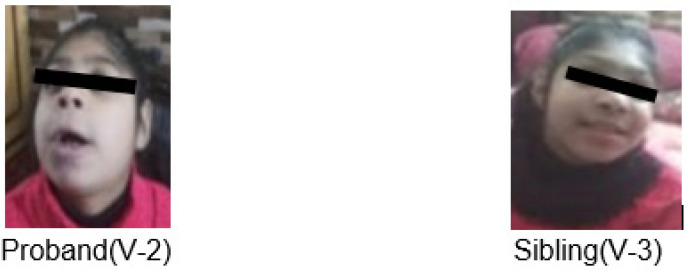
Case presentation and photographic images of syndromic MCPH phenotypes in proband and affected sibling.

**Figure 2:**
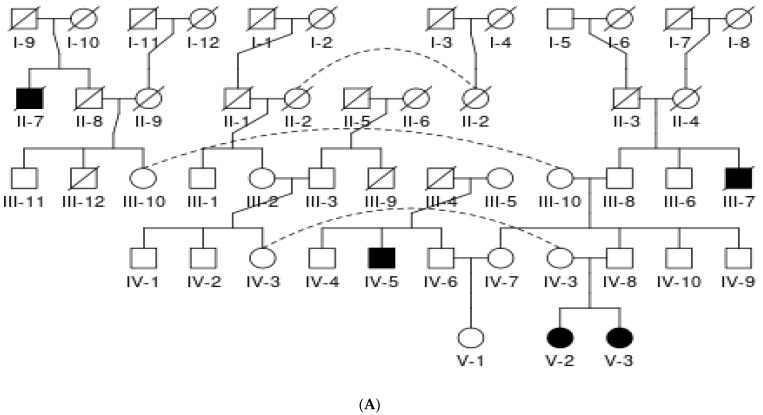

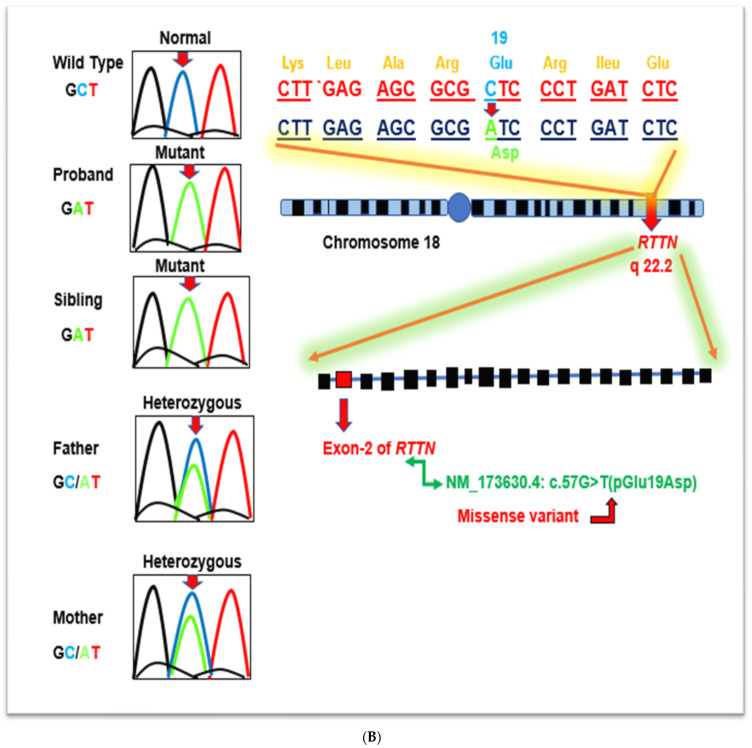
(**A**) Pedigree of the affected family showing inheritance pattern in five successive generations. Filled shapes show disease-affected members, circle represents female, square represents male, double marriage line shows consanguinity, diagonal line represents deceased and black arrow is pointed towards proband. (**B**) *RTTN* gene variant c.57G > T(pGlu19Asp) of chromosome 18 in exon 2 of proband. Sanger sequencing results in the family members showing co-segregation analysis.

A 10-year-old girl was referred to a neurologist at OPD Neurology, Pakistan Institute of Medical Sciences hospital, Islamabad, for proper diagnosis of congenital microcephaly and seizures. She had a positive family history as her 8-year-old sister shared the same disorder (Table 1). The patient phenotype shows short stature, slim body with distorted facial features such as a narrow forehead, bulging eyes, pointed nose, fuller lips, broad mouth, and constant drooling, while her ophthalmic exam reported reduced vision due to retinal lesions and bilateral cataracts. She had good control of her head and other body parts, socially recognized her parents and was smiling. Both children faced developmental delays, learning disabilities, repetitive behavior, absence of speech and mood disturbance. The patient had an MRI exam that showed frontal lobe disproportion, cortical simplification, and myelin dystrophy. Her mother reported no viral infection or medication during full-term pregnancy and normal delivery. An in-depth examination confirmed the clinical diagnosis of primary microcephaly, and the family was referred to genetics for whole-exome sequencing (Figure 1 and Figure 2A).

### 2.2. Genetic Analysis

The family contacted the human molecular genetics research group at the Translational Genomics Lab, COMSATS University, Islamabad, for genetic analysis of the phenotype (in January 2022). The parents were first cousins and both daughters suffered from autosomal recessive primary microcephaly syndrome. Pedigree analysis was performed for five generations and DNA from the proband, affected sister and normal parents was extracted from a 2 mL venous blood sample via the phenol-chloroform method. DNA was shipped to Macrogen Inc., Korea, for whole-exome sequencing. The received VCF files were uploaded to Franklin Genoox software for pathogenic variant identification. The results of whole-exome sequencing analysis uncovered that the syndromic MCPH phenotype is due to a missense variant in exon 2 of the *RTTN* gene (NM_173630.4: c.57G > T(pGlu19Asp), classified as pathogenic and deleterious by Franklin Genoox software and in silico bioinformatics tools SIFT, Polyphon2, Mutation Taster, Mutation Assessor and FATHMM (accessed June 2022). Evaluation of the variants was performed by the American College of Medical Genetics (ACMG) criteria. The frequency of the alleles was examined in control population databases that include 1000 Genomes, gnomAD and ESP6500 (Table 2). Sanger sequencing was performed for validation of the genetic mutation and its co-segregation analysis in the affected and unaffected family members. Sanger sequencing data analysis (by Finch TV; accessed August 2022) detected the causative variant in the proband and affected sibling as biallelic, while the parents were heterozygous (Figure 2B).

### 2.3. Functional Analysis

**Figure 3:**
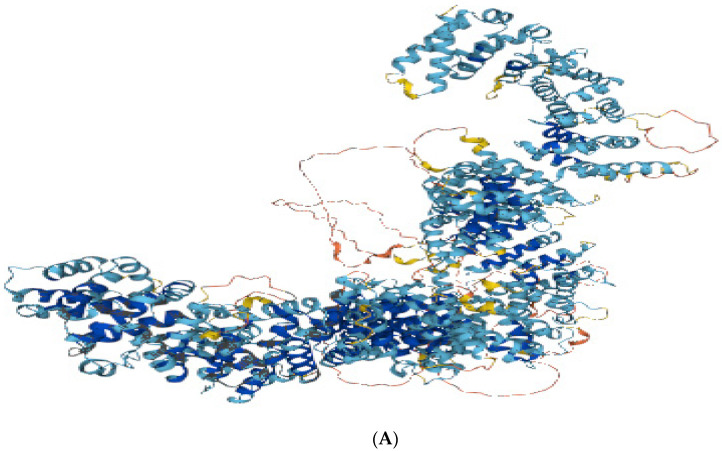

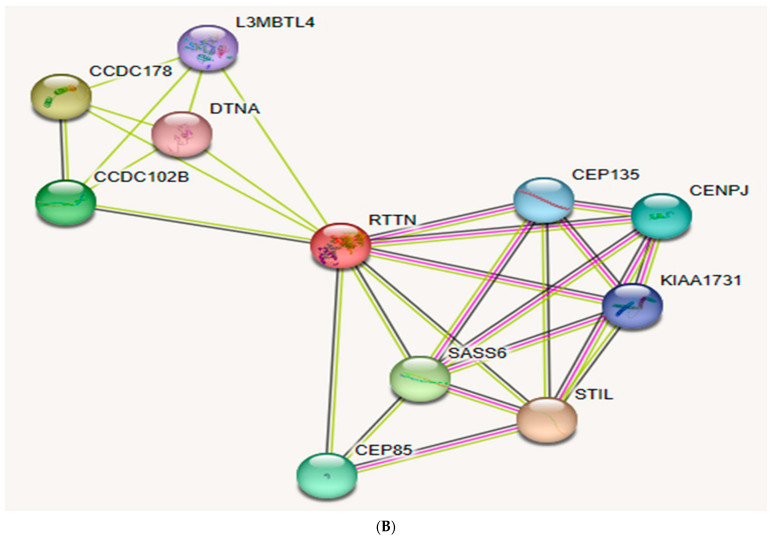
(**A**) Three-dimensional RTTN protein model (UniProt Database) with blue color showing a high confidence score for protein residues, while rust color represents the residues, which may be unstructured due to low confidence score per residue, as predicted by alpha-Fold. (**B**) RTTN protein–protein functional enrichment analysis using STRING database depicts the role of STIL (centriole duplication), SASS6 (spindle assembly protein ensuring their symmetry), CEP85 (maintains centrosome integrity during interphase stage), CENPJ (centriole duplication during cell-cycle), CEP135 (centriole biogenesis), KIAA1731 (centriole-to-centrosome conversion at late mitosis), L3MBTL4 (chromatin modification) and DTNA (formation and stability of synapses) proteins in expression pathways of human microcephaly protein rotatin, which essentially governs genetic cascade of normal ciliary structure, centriole biogenesis, orientation of mitotic spindles and organization of cerebral cortex in human embryos. In our analysis, PPI enrichment *p*-value (3.48 × 10^−6^) predicts significant interactions and those that are more than expected in terms of biological and cellular processes (gene ontology), including centriole elongation, replication, duplication, centriole–centriole cohesion, assembly and cell-cycle process regulation that predicts the RTTN protein role in neural pathophysiology of familial microcephaly. **Legend:** Empty nodes represent proteins of unknown 3D structure, while filled nodes are for known 3D protein structures. Green connecting lines represent the known interactions from curated databases, while purple lines show experimentally determined interactions. Red connecting lines represent gene fusions, and blue lines represent gene co-occurrence, while black lines represent the co-expression of genes in protein networks. Colored nodes are query proteins with the first line of interaction. The number of nodes is 11, number of edges is 28, expected number of edges is 10, average node degree is 5.09 and average local clustering co-efficient is 0.909.

**Figure 4:**
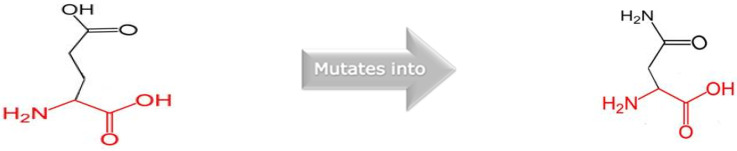
The wild type of amino acid residue was part of the alpha helix of protein secondary structure; this functional interaction was lost for the mutated residue, and the affected protein was predicted to be deleterious for the protein function.

Mutations in *RTTN,* which codes for centrosome-associated proteins, cause cell-cycle anomalies and cortical malformations expressed as a spectrum of neurological and developmental clinical phenotypes. The multifunctional and dynamic pre-mitotic characteristics of the RTTN protein are still to be explored. The protein model was generated for the mutated variant (HOPE prediction tool), and analysis of wild type and mutated amino acid residue was performed. Mutated amino acid residue was decreased in size with a neutral charge, as compared to wild type which was bigger and negatively charged. HOPE characterized the mutation as damaging to the protein structure and function (Figure 3 and Figure 4).

## 3. Discussion

We present a novel, missense variant in the *RTTN* gene NM_173630.4: c.57G > T(pGlu19Asp) that changes the amino acid sequence in the resulting protein, making it functionally pathogenic. Seckel syndrome is a very rare and challenging neurological impairment that results in reduced brain dimensions and severe intellectual disability due to microcephaly, along with short stature [20]. Recently, multiple genetic studies have reported the probable role of *RTTN* variants in microcephaly syndromes [21]. *RTTN* expression pathways significantly control mitotic proliferation and modify spindle orientation in the cell-cycle [22]. Loss-of-function missense mutations in *RTTN* gene cause mitotic aberrations leading to autosomal recessive syndromic phenotypes [23].

The NCBI database reports 46 exons in the *RTTN* gene which are expressed as rotatin protein. Homozygous and deleterious variations in the *RTTN* gene have been associated with a number of neurological conditions, including microcephaly, and extracranial defects [24]. The *RTTN* gene in humans was reported for the first time in 2012 with a homozygous mutation in three members of a family in Turkey who had a consanguineous marriage, and offspring had short height, epilepsy, microcephaly and polymicrogyria [23,25].

Seckel syndrome has a variable expression associated with primary microcephaly, dwarfism, specific facial features and ophthalmic manifestations, as observed in our patient [26,27]. Seckel syndrome cases are often misdiagnosed, especially when patients do not exhibit all the phenotype landmarks and resemble other syndromes. The main difference between Seckel syndrome growth retardation and other growth retardation syndromes is its feature of proportionate growth arrest [28,29]. Microcephalic dwarfism has been linked to ophthalmic manifestations such as glaucoma and retinal lesions, as reported in many cases previously, which is in strong agreement with our case study [30]. Case-to-case clinical diagnosis and genetic identification is required and seems appropriate for reporting data for co-existing pathological conditions [31]. Polymicrogyria, a cortical deformity, was first linked to recessive mutations in the *RTTN* gene. *RTTN* mutations have been linked to a wide range of additional brain abnormalities, including primary microcephaly. We were able to extrapolate the main characteristics, which included intellectual disability, short stature, microcephaly, lissencephaly, cardiac abnormalities, polymicrogyria, cataract and other malformations, from the published clinical cases and genomic variants, including the effect of novel exonic and intronic pathogenic mutations in the *RTTN* gene. We show that the severity of the condition is predicted to be connected to the protein’s residual function, not just the quantity of mRNA expression [32]. Thus, the neurodevelopmental and extra-neurological features in our patient are confirmed by reviewing previously available literature data and findings for the *RTTN* gene [33].

Cellular and molecular pathways implicating MCPH genes elucidated the role of protein mutations affecting the centrosome and spindle fiber biogenesis, their localization and orientation that actually determine the brain size and dimensions [34]. The main genes [10] reported for cell cycle progression, centriole biogenesis, centrosome orientation, microtubule assembly and regulation of kinases pathways for phosphorylation are involved in microcephaly syndromes [35]. Familial autosomal recessive microcephaly in Seckel syndrome cases are mainly observed as a result of truncated protein complexes formed due to missense and frameshift variants in *ATR*, *RBBP8*, *CENPJ*, *NSMCE2*, *DNA2*, *NIN*, *CEP152*, *CEP63* and *TRAIP* [36]. Recent researches have determined the role of novel variants in NUP85, *CRIPT* and *CEP63* genes expressed as primordial dwarfism and microcephaly in Seckel syndrome case reports [1,37,38]. Our study has expanded this spectrum by adding a novel biallelic loss-of-function variant in the *RTTN* gene associated with neurological malformation, congenital deformities, and growth abnormalities.

There is still much to understand about the specific molecular pathways that orchestrate appropriate cortical development and brain growth. However, significant progress in this field has been made through genetic studies of inherited forms of primary microcephaly and cortical development malformations. A review of all known *RTTN* mutations reveals that they are not constrained to specific places or suspected rotatin functional domains, nor do they correlate with indications [39]. Exonic missense variants of the *RTTN* gene have recently been reported and linked to the severity of phenotype features of Seckel syndrome [32]. Finally, our reported case presentation of *RTTN* mutation is a complicated human disease phenotype. Our study elaborates that the rotatin protein is predicted to be involved in the advancement of cell cycle, replication, and neural movement, offering insights into Seckel syndrome traits, and establishing its crucial role within the ever-evolving system of centrosome genes.

## 4. Conclusions

We highlighted a novel pathogenic missense variant affecting *RTTN* gene expression as a contribution to the diagnosis of MCPH. A literature review shows few cases reported to date for autosomal recessive congenital microcephaly with short stature, bilateral cataract, absence of speech and prominent facial features diagnosed as Seckel syndrome [18]. Our case presentation is unique as two siblings showed the same neurological features who were born to normal parents, and their MRI depicts myelin dystrophy in addition to conventional disease landmarks. Further investigations are needed for the validation of the causative variant and underlying pathophysiological disease mechanisms. Here, we have made a significant contribution to the knowledge, in the hope that better clinical care and genetic counselling for the family may prevent more births with same disorder.

## Figures and Tables

**Table 1:** Phenotype landmarks of Seckel syndrome patients.

Patient ID	Age	Gender	Onset Age	Primary Condition	Associated Conditions	Clinical Diagnosis
Proband V-2	10 years	F	Congenital	MCPH Short	ID, Seizures, Cataract	Seckel Syndrome Suspected
V-3	8 years	F	Congenital	MCPH Short	ID, Seizures, Cataract	Seckel Syndrome Suspected

**Table 2:** WES data for proband with pathogenic variant.

Gene	Chromosome	Ref	Alt	Transcript (Exon)	Nucleotide Change	AA Change	Variation Type (Effect)
*RTTN*	18	C	A	NM_173630.4 (2)	c.57G > T	pGlu19Asp	Missense

## Data Availability

Data are available on request from the authors.

## Abbreviations

MCPH	Microcephaly Primary Hereditary
ID	Intellectual Disability
DD	Developmental Delay
OPD	Outpatient Department
WES	Whole-Exome Sequencing
